# Dysregulated noradrenergic response is associated with symptom severity in individuals with schizophrenia

**DOI:** 10.3389/fpsyt.2023.1190329

**Published:** 2023-11-07

**Authors:** Ana Pelegrino, Anna Luiza Guimaraes, Walter Sena, Nwabunwanne Emele, Linda Scoriels, Rogerio Panizzutti

**Affiliations:** ^1^Instituto de Psiquiatria, Universidade Federal do Rio de Janeiro, Rio de Janeiro, Brazil; ^2^Instituto de Ciências Biomédicas, Universidade Federal do Rio de Janeiro, Rio de Janeiro, Brazil; ^3^Université Paris Cité, Institut de Psychiatrie et Neurosciences de Paris, Inserm, Paris, France

**Keywords:** schizophrenia, cognition, symptom severity, locus coeruleus, noradrenergic response, pupil dilation

## Abstract

**Introduction:**

The locus coeruleus-noradrenaline (LC-NA) system is involved in a wide range of cognitive functions and may be altered in schizophrenia. A non-invasive method to indirectly measure LC activity is task-evoked pupillary response. Individuals with schizophrenia present reduced pupil dilation compared to healthy subjects, particularly when task demand increases. However, the extent to which alteration in LC activity contributes to schizophrenia symptomatology remains largely unexplored. We aimed to investigate the association between symptomatology, cognition, and noradrenergic response in individuals with schizophrenia.

**Methods:**

We assessed task-evoked pupil dilation during a pro- and antisaccade task in 23 individuals with schizophrenia and 28 healthy subjects.

**Results:**

Both groups showed similar preparatory pupil dilation during prosaccade trials, but individuals with schizophrenia showed significantly lower pupil dilation compared to healthy subjects in antisaccade trials. Importantly, reduced preparatory pupil dilation for antisaccade trials was associated with worse general symptomatology in individuals with schizophrenia.

**Discussion:**

Our findings suggest that changes in LC-NA activity – measured by task-evoked pupil dilation – when task demand increases is associated with schizophrenia symptoms. Interventions targeting the modulation of noradrenergic responses may be suitable candidates to reduce schizophrenia symptomatology.

## 1. Introduction

Schizophrenia symptoms may arise from dysregulation in neuromodulatory systems. Evidence suggests that the locus coeruleus-noradrenergic (LC-NA) system might be involved in the development of schizophrenia pathophysiology (1, 2), although the prevailing hypothesis involves the dopaminergic and glutamatergic systems (3, 4). Cognitive deficits, for example, have long been recognized as a core feature of schizophrenia and are associated with poor functional outcomes (5–7). There has been a growing interest in studying cognitive deficits in schizophrenia as they predict functional outcomes, resist antipsychotic treatment, and often persist throughout life (7–11).

The LC-NA system plays a pivotal role in regulating cognitive functions, such as working memory, learning and attention, and memory consolidation (12–17), which are largely impaired in schizophrenia. In individuals with schizophrenia, noradrenaline (NA) is elevated in both the blood and the cerebrospinal fluid (CSF) (18), especially in those with positive symptoms (e.g., delusions, hallucinations, thought disorders) (19). Postmortem studies have also reported increased markers for NA in the brains of schizophrenia individuals (20, 21). In general, studies associated positive symptoms with hyperactivity of the NA system, while negative symptoms (e.g., affective blunting, inattention, abulia) were related to hypoactivity of the NA system (1). For instance, diminished frontocentral P300 amplitudes observed in individuals with schizophrenia during oddball and inhibition tasks may be an indicator of LC hypoactivity (22). Additionally, drugs increasing the central NA concentration (e.g., modafinil) lead to better performance in some cognitive abilities (23, 24).

Task-evoked pupil dilation is a non-invasive, well-established indirect measure of LC-NA activity (12, 25–27) and has been used as an effective indicator of cognitive processing (28–32). For example, pupil dilation scales with levels of difficulty across a variety of cognitive tasks (e.g., N-back, Digit Span, Stroop tasks), and it is associated with task difficulty in different cognitive domains (28, 33–40). Among healthy subjects, changes in pupil size during the preparatory period to exert an antisaccade, which requires a saccade to the opposite side of the target, are larger than for prosaccades (41).

In cognitive tasks, individuals with schizophrenia tend to exhibit reduced pupil dilation compared to healthy subjects, especially as task demands increase (39, 42–45). For instance, reduced task-evoked pupil response in preparation for antisaccades might contribute to deficits in executive cognitive control in individuals across the psychosis spectrum (46). Interestingly, changes in pupil size for pro-and anti-saccades were reported as similar (47), which may suggest a deficit in LC activity regulation in response to higher task demands in schizophrenia. Moreover, reduced pupil dilation has been related to the severity of negative symptoms of schizophrenia (44, 48). For example, greater pupil dilation was associated with worse motivational negative symptoms, which include lack of motivation, anhedonia, and asociality (49). Additionally, in the digit span task, a measure of working memory capacity, individuals with schizophrenia who had severe defeatist attitudes showed significantly less pupil dilation when processing demands increased compared to individuals with mild defeatist attitudes (43). Despite the groundwork in this area, the relationship between LC-NA system dysregulation and schizophrenia symptomatology is still not fully comprehended.

In the present study, we evaluated whether impairments in the LC-NA system, measured using task-evoked pupil dilation, were associated with impairments in the performance of cognitive tasks and clinical symptoms in individuals with schizophrenia compared with matched healthy subjects. First, we examined participants’ task-evoked pupillary response using an oculomotor task that required lower cognitive effort (i.e., prosaccade) and higher cognitive effort (i.e., antisaccade). Considering previous findings, we expected that individuals with schizophrenia would exhibit reduced pupil dilation in the antisaccade task compared to healthy subjects. Second, we examined the relationship between task-evoked pupil dilation, cognition and clinical symptoms. We expected that abnormal pupillary response would be associated with cognitive deficits and increased general symptomatology.

## 2. Methods

### 2.1. Participants

The sample included 23 subjects with chronic schizophrenia or schizoaffective disorder recruited from the day-hospital and outpatient clinic of the Institute of Psychiatry (IPUB) at the Federal University of Rio de Janeiro, from September 2013 to December 2019.

The diagnosis was established by a board-certified psychiatrist using a best estimate approach, combining information from medical records and the results of the Structured Clinical Interview for DSM-IV in a validated Brazilian Portuguese version. Chlorpromazine (CPZ, antipsychotic medication) (50) and Benztropine (anticholinergic medication) (51) equivalents (mg) were calculated (Table 1). All patients were stable in the same medication during the period of the experiment and had not suffered a psychotic relapse in the 2 months before study entry. IQ was estimated from the WAIS-III Brazilian version and the severity of symptoms from the Positive and Negative Syndrome Scale (PANSS). Participants were included if they (1) were between the ages of 18 and 65 years of age, (2) had a diagnosis of schizophrenia or schizoaffective disorder, (3) were clinically stable, and had an outpatient status for at least 2 months before starting the study, and (4) had Portuguese as the primary language. Participants were excluded if they (1) had any history of another psychiatric diagnosis, (2) history of psychotic episodes in the previous 2 months, (3) had intellectual disability (IQ < 80), (4) diagnosis of substance dependence, and (5) uncorrected visual or hearing problems.

**Table 1:** Participants’ characteristics.

	Individuals with schizophrenia	Healthy subjects	
	*N* = 23	*N* = 28	
	Mean (SD)	Mean (SD)	Statistics^a^
Male | female	14 | 10	18 | 10	X^2^ = 0.19, *p* = 0.66
Age (years)	37.39 (12.12)	36.93 (11.38)	*t* = 0.09, *p* = 0.92
Education (years)	10.67 (2.61)	11.24 (2.53)	*t* = 0.81, *p* = 0.42
Years of illness	15.96 (12.31)	NA	
IQ	102.7 (14.29)	NA	
Clinical symptoms (30–210)			
PANSS total score	55.30 (11.37)	NA	
PANSS positive score	12.22 (5.56)	NA	
PANSS negative score	14.52 (5.42)	NA	
PANSS general score	28.39 (5.53)	NA	
Cognition (z-scores)			
Global cognition	−0.91 (0.98)	NA	
Attention	−1.72 (1.76)	NA	
Speed of processing	−1.33 (1.85)	NA	
Working memory	−1.17 (1.54)	NA	
Visual memory and learning	−0.25 (0.86)	NA	
Verbal memory and learning	−0.34 (0.98)	NA	
Reasoning and problem solving	−0.54 (1.00)	NA	
Social cognition	−1.12 (1.85)	NA	
Performance			
Antisaccade error rate	0.38 (0.13)	0.30 (0.20)	*t* = 1.78, *p* = 0.08
Antisaccade RT (ms)	242.5 (57.70)	240.3 (63.78)	*t* = 0.12, *p* = 0.90
Prosaccade RT (ms)	**203.6 (41.46)**	**170 (36.05)**	***t* = 3.01, *p* = 0.003**
Medication (mg)			
CPZ equivalent	374.7 (263.8)	NA	
Benztropine equivalent	2.92 (3.42)	NA	

a Chi-squared test for sex and Independent Samples t-test (two-tailed) for age and education. IQ, intelligence quotient. PANSS, Positive and Negative Syndrome Scale. RT, reaction time. CPZ, chlorpromazine. Benztropine equivalent was calculated for 21 subjects.

For the control group, a board-certified psychiatrist recruited 28 volunteers via personal approach. They were free from current or past history of major psychiatric illness and denied a family history of psychotic disorders in first-degree relatives. Recruitment of healthy controls was managed to match the schizophrenia group by sex, age, education, and ethnicity.

All participants signed a written consent form after being informed about the study procedures. The study was approved by the Brazilian National Committee of Ethics in Research (12990013.0.0000.5263) and pre-registered at ClinicalTrials.gov (1R03TW009002–01).

### 2.2. Assessments

#### 2.2.1. Cognition

We measured the performance of participants in seven cognitive domains that have been defined as impaired in schizophrenia by the Measurement and Treatment Research to Improve Cognition in Schizophrenia (MATRICS). We used the following tests: the Cambridge Neuropsychological Test Automated Battery (CANTAB) Reaction Time (RT) and Cogstate Detection to assess speed of processing; the CANTAB Rapid Visual Processing (RVP) and the Cogstate Identification to evaluate attention; the CANTAB Spatial Working Memory (SWM) and Cogstate Two Back for working memory; the MATRICS Consensus Cognitive Battery recommended tests (MCCB) Hopkins Verbal Learning Task (HVLT) for verbal learning; the MCCB Brief Visuospatial Memory Test for visual learning; the CANTAB Stocking Of Cambridge (SOC) and Cogstate Groton Maze Learning for reasoning and problem-solving; and the MCCB Mayer-Salovey-Caruso Emotional Intelligence Test for social cognition. Raw scores were converted to z-scores using developer normative data. Further details regarding cognitive assessments are available in our previous work (9).

#### 2.2.2. Symptoms

The PANSS was administered to assess participants’ clinical status (52).

### 2.3. Recording and apparatus

Eye position and pupil size were measured with a video-based eye tracker (Eyelink-1000 plus; SR Research Ltd., Ontario, Canada) at a rate of 1,000 Hz with a monocular recording (the right eye and pupil were used). After a 9-point calibration, stimuli were presented on a 23-inch LCD monitor (Philips 144 Hz refresh rate and 1920×1080 pixels resolution). The distance from the eyes, with the head on a chin rest, to the monitor was 56 cm. The pupil diameter provided by EyeLink gives pupil area or diameter in arbitrary units. Therefore, we converted the arbitrary units into absolute units, that is, millimeters (see Supplementary material). All participants were tested under the same lighting conditions.

### 2.4. Oculomotor task

**Figure 1:**
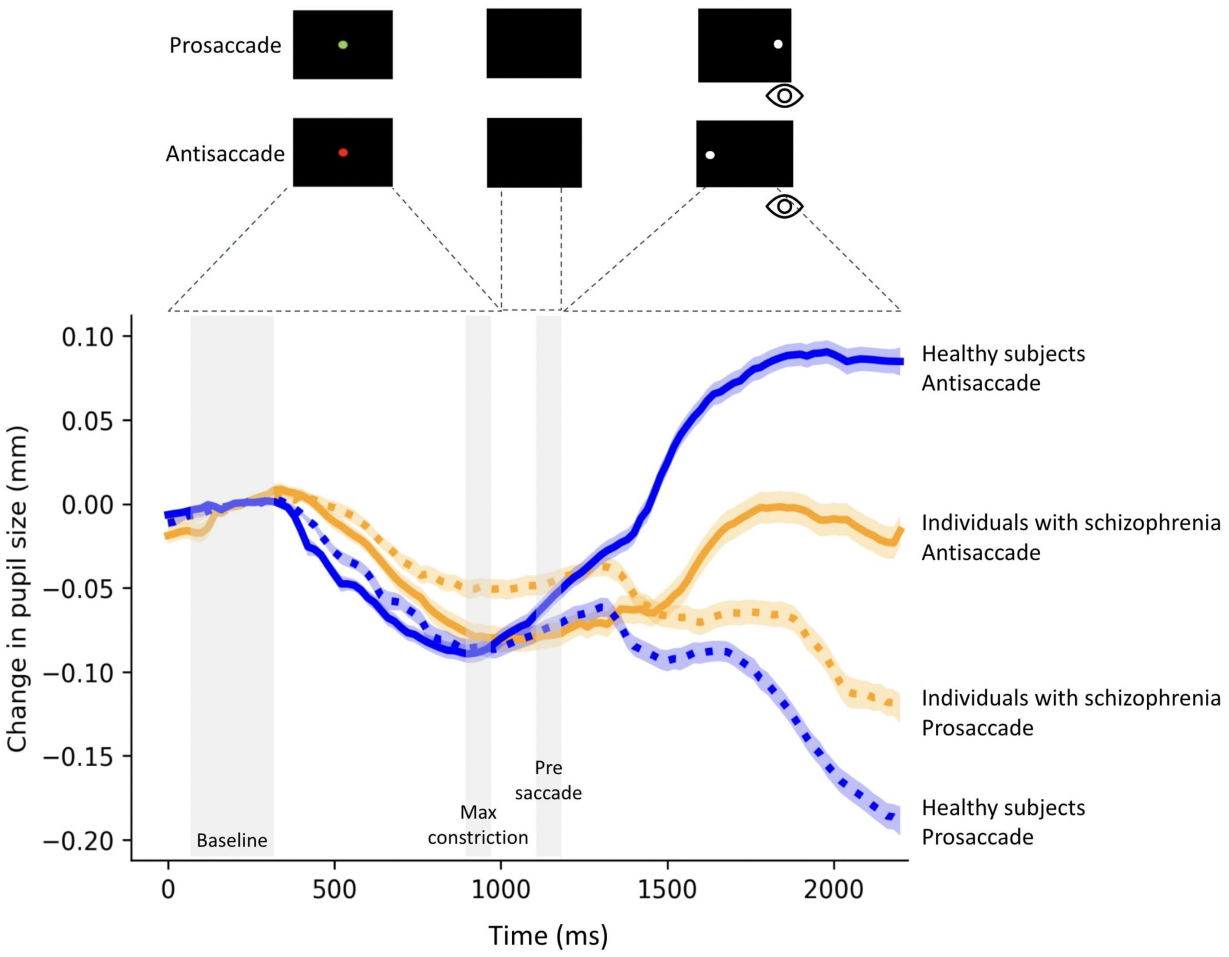
Change in pupil size (baseline-corrected to 150-300 ms of fixation point onset) for correct prosaccade and antisaccade trials in 23 individuals with schizophrenia and 28 healthy subjects. The shaded colored regions around the pupillary response represent +/− standard error range (between participants) for the different conditions. The gray area represents the selected epochs for pupil analyses. The magnitude of pupil dilation during task preparation was calculated by averaging the pupil size during the Pre-saccade epoch (50 ms before peripheral stimulus onset) minus the average pupil size over 50 ms at the time of maximum constriction (Max constriction) during fixation.

Participants performed the prosaccade and antisaccade tasks. Each trial began with the appearance of a green (prosaccade condition) or red (antisaccade condition) fixation point (FP) (diameter, 0.5°; 42 cd/m2) on a black background (0.1 cd/m2). In prosaccade trials, participants were instructed to look toward the peripheral stimulus and in the opposite direction in antisaccade trials. After 1,000 ms of central fixation, the FP disappeared for 200 ms before the peripheral stimulus appeared for 1,000 ms (diameter, 0.5°; white dot with luminance 42 cd/m2) to the left or right of the FP (10° eccentricity on the horizontal axis) (Figure 1). Each trial had an inter-trial period of 1,000 ms which provided enough time for the pupil to return to baseline. The experiment consisted of 60 trials of pro-and 60 trials of antisaccade. Trial conditions (prosaccade and antisaccade) and stimulus location (left and right) were randomly interleaved.

Reaction time (RT) was defined as the time from response cueing to saccade onset, determined when eye velocity exceeded 30°/s. Trials were correct if the first saccade after the FP was in the correct direction. Directional errors were identified as the first saccade after FP that was executed in the wrong direction (e.g., toward stimulus on antisaccade trials). The error rate was calculated as the ratio of the number of incorrect antisaccades to the total number of antisaccades. Trials with RT less than 90 ms were excluded from the data analysis (53).

For pupil data, trials with an eye position deviation of more than 2° from the FP during the period of central fixation were excluded from the analysis. If the eye is in a blink, the EyeLink will report the specific field as missing values. Therefore, when blinks were detected, following the literature, pre-, and post-blink pupil values were used to do linear interpolation to replace values during the blink period (47). Importantly, there was no significant difference between groups for the number of detected blinks (prosaccade: *t* = 1.30, *p* = 0.20; antisaccade: *t* = 0.21, *p* = 0.82). Only correct trials were used for the analyses.

Approximately 30% of trials were excluded from the analysis. At least 53% of trials were included for statistical analysis. At least 22 trials remained for each condition from each participant. Importantly, there was no significant difference between groups for the total trials excluded (*t* = 0.83, *p* = 0.40).

We evaluated relative pupil size using baseline correction (41, 54). The baseline pupil diameter value was determined by averaging pupil size from the first 150-350 ms after fixation onset, and for each trial, original pupil diameter values were subtracted from this baseline pupil diameter value. As video-based tracking systems can distort pupil size following variations in eye gaze (55), we examined pupil size prior to saccade initiation. Following previous work (41), pupil size was determined in three epochs prior to saccade initiation (i.e., when the gaze was located at the center of the screen): the start of the fixation point epoch (150-300 ms after fixation onset), the maximal pupil constriction, and the pre-saccade epoch (50 ms before peripheral stimulus onset) (Figure 1).

The change in pupil size prior to saccade initiation was defined as the averaged pupil size during the pre-saccade epoch *minus* the averaged pupil size across 50 ms at the time of greatest constriction during fixation.

### 2.5. Statistics

The distributions of demographic, cognitive, clinical, and pupil size data were tested for normality using the Shapiro–Wilk Test. Independent Samples t-tests (two-tailed) or Chi-squared tested for group differences in demographic variables between healthy subjects and individuals with schizophrenia. We performed a mixed ANOVA (2×2 ANOVA: between-subjects factor: individuals with schizophrenia/healthy subjects x within-subjects factor: (prosaccade/antisaccade)) for statistical analysis. We further included the results from the dependent and independent t-tests as a *post-hoc* analysis to specifically test our hypothesis that the modulation of pupil size (prosaccade x antisaccade) was impaired in the schizophrenia group, but intact in the healthy control group. First, within-group post-hoc comparisons between changes in pupil size for prosaccades and antisaccades were calculated using Paired samples t-tests (2-tailed). Second, between-groups post-hoc comparisons were calculated using Independent samples t-tests (2-tailed). Pearson or Spearman correlations were used to examine associations between the change in pupil size in prosaccades and antisaccades, cognition, clinical symptoms, performance, and medication in the schizophrenia and control group.

We used participants’ neuropsychological tests z-scores and the raw data of the clinical scales. Data were analyzed using IBM SPSS (28.0 version) with a statistical significance level set at *p* < 0.05.

## 3. Results

### 3.1. Participants

Participants’ characteristics are presented in Table 1. The two groups did not differ in any demographic characteristics.

### 3.2. Performance in prosaccade and antisaccade trials

Participants’ performance was evaluated using antisaccade reaction time (RT) and error rate. As expected, individuals with schizophrenia and healthy subjects had significantly longer RTs for antisaccade than for prosaccade trials (*t* = 5.74, *p* < 0.0001; *t* = 6.62, *p* < 0.0001 respectively). Given previous findings of longer RTs and higher direction errors in schizophrenia in the antisaccade task (56), antisaccade RTs and antisaccade error rates were compared between groups. We found a trend toward significance for individuals with schizophrenia to make more errors than controls (healthy subjects: mean = 0.30, SD = 0.20; individuals with schizophrenia: mean = 0.38, SD = 0.13; *t* = 1.78, *p* = 0.08). No significant differences between groups were observed for antisaccade RT (healthy subjects: mean = 240.3 ms, SD = 63.78 ms; individuals with schizophrenia: mean = 242.5 ms, SD = 57.70 ms; *t* = 0.12, *p* = 0.90). Interestingly, individuals with schizophrenia showed significantly longer prosaccade RT than healthy subjects (mean = 203.6 ms, SD = 41.46 ms; mean = 170 ms, SD = 36.05 ms, respectively; *t* = 3.01, *p* = 0.003).

### 3.3. Pupillary responses for correct prosaccade and antisaccade trials

Figure 1 shows relative pupil diameter corrected by the diameter at fixation onset (see methods), revealing two components of the pupil response: an initial constriction that began shortly after fixation point appearance followed by pupil dilation. The initial constriction is mainly driven by the changes in luminance level following the presentation of a luminant fixation point, while the dilation is related to task preparation (41).

Baseline pupil size was similar between schizophrenia and healthy subjects (prosaccade (*t* = 0.60; *p* = 0.55)), antisaccade trials (*t* = 0.50; *p* = 0.61). There was also no significant difference in pupil size at baseline between trial conditions in individuals with schizophrenia (*t* = 0.1.36; *p* = 0.18) and healthy subjects (*t* = 0.18; *p* = 0.85).

### 3.4. Pupil dilation in task preparation

**Figure 2:**
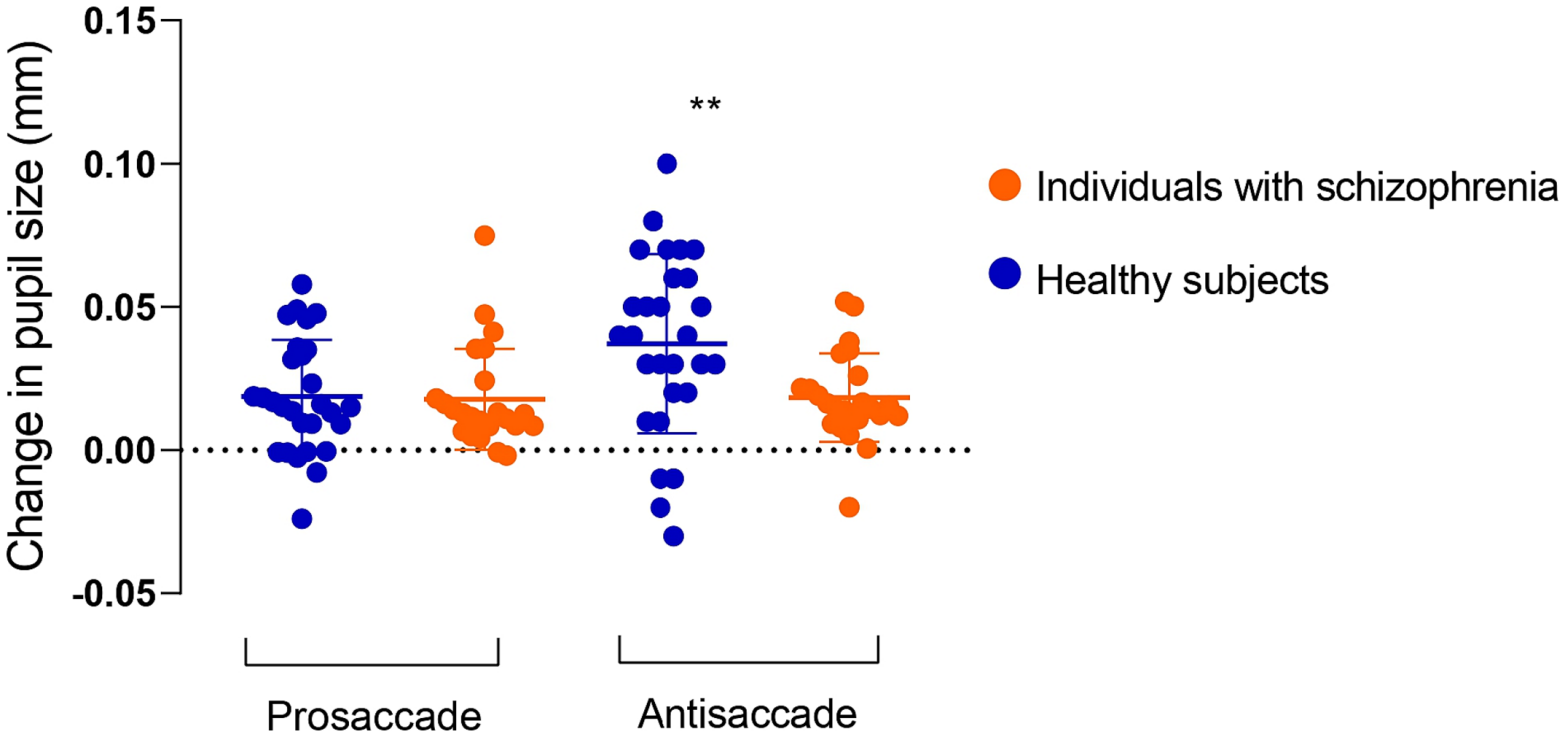
Change in pupil size in preparation for correct prosaccade and antisaccade in 23 individuals with schizophrenia and 28 healthy subjects. Pupil dilation in antisaccade preparation was reduced in individuals with schizophrenia compared to healthy subjects. Values are presented as mean and standard deviation.

Figure 2 outlines pupil dilation prior to saccade initiation in prosaccade and antisaccade trials in healthy subjects and individuals with schizophrenia. The effect of trial (prosaccade/antisaccade) and group on pupil dilation was assessed using a mixed model ANOVA. There was a significant main effect of trial condition (*F* (1, 49) = 9.90, *p* = 0.003, η (2)= 0.16), trial-by-group interaction (*F* (1, 49) = 8.16, *p* = 0.006, η (2)= 0.14) and a trend towards significance for group (*F* (1, 49) = 3.27, *p* = 0.07, η (2)= 0.06). Within-group post-hoc comparisons revealed larger pupil dilation in antisaccade preparation compared to prosaccade preparation in healthy subjects (prosaccade mean = 0.018 mm, SD = 0.019; antisaccade mean = 0.037 mm, SD = 0.031; *t* = 3.82, *p* < 0.0007; Hedges’s g = 0.68), but not in individuals with schizophrenia (prosaccade mean = 0.017 mm, SD = 0.018; antisaccade mean = 0.018 mm, SD = 0.015; *t* = 0.26, *p* = 0.26). Between-group post-hoc comparisons revealed reduced pupil dilation in individuals with schizophrenia compared to healthy subjects (*t* = 2.62, *p* = 0.01; Hedges’ g = 0.72). In contrast, pupil dilation during prosaccade preparation was similar between schizophrenia and healthy controls (*t* = 0.23, *p* = 0.81). Importantly, all statistics were maintained even when blinks were removed from the analysis (Supplementary material).

**Figure 3:**
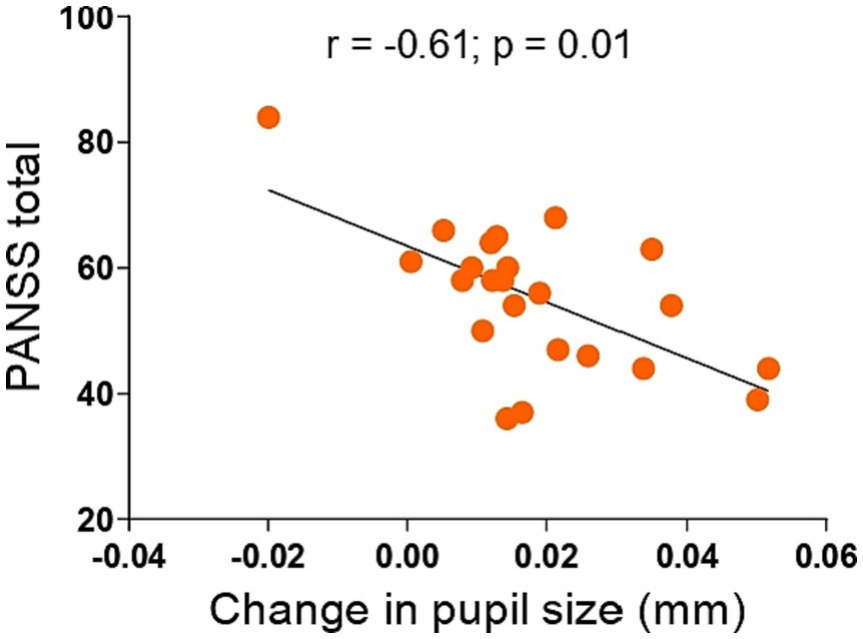
Associations between change in pupil size in antisaccade preparation and PANSS total in 23 individuals with schizophrenia. Smaller pupil dilation in antisaccade preparation was associated with higher PANSS total scores. Inserts show r and *p* values of the Spearman correlation (2-tailed). PANSS, Positive and Negative Syndrome Scale.

Next, we examined if the pupil size increase during antisaccade preparation would be associated with cognition, symptoms, or medications. We found no significant associations between pupil dilation in antisaccade preparation and cognition (data not shown). In regards to symptomatology, lower pupil dilation was negatively correlated with PANSS total (*r* = −0.61; *p* = 0.01) score (Figure 3; Table 2) and, at a trend level, with PANSS general (*r* = −0,39, *p* = 0.06) and negative (*r* = −0.36, *p* = 0.08) scores (Table 2); this result indicates that, for individuals with schizophrenia, the higher the symptomatology scores, the lower the pupil size will increase in preparation for antisaccade trials.

**Table 2:** Associations between change in pupil size in antisaccade preparation, cognition, clinical symptoms, performance, and medication in individuals with schizophrenia.

	Change in pupil size (mm)		
	*r*	*p*	*N*
Cognition (z-scores)			
Global cognition	0.20	0.34	23
Attention	0.22	0.28	23
Speed of processing	0.24	0.25	23
Working memory	0.18	0.39	23
Visual memory and learning	0.06	0.77	23
Verbal memory and learning	−0.07	0.72	23
Reasoning and problem solving	0.14	0.49	23
Social cognition	0.27	0.25	21
Clinical symptoms			
PANSS total score	**−0.61**	**0.001**	**23**
PANSS positive score	−0.29	0.17	23
PANSS negative score	−*0.36*	*0.08*	*23*
PANSS general score	−*0.39*	*0.06*	*23*
Performance			
Antisaccade error rate	**−0.55**	**0.005**	**23**
Antisaccade RT (ms)	−0.03	0.88	23
Medication (mg)			
CPZ equivalent	−0.13	0.54	23
Benztropine equivalent	−0.15	0.50	22

PANSS, Positive and Negative Syndrome Scale. CPZ, chlorpromazine. RT, reaction time. *r* and *p* values of the Pearson or Spearman correlations (2-tailed). N, number of subjects. Bold values show *p* values <0.05. Italic numbers trend towards significance (0.05 < *p* < 0.1).

We also examined the relationship between pupil dilation and task performance across subjects, as indexed by error rate and RTs. We found a negative correlation between pupil dilation in antisaccade preparation and antisaccade error rate in individuals with schizophrenia (*r* = −0.55, *p* = 0.005), but not in healthy subjects (Table 2). No significant correlation between pupil dilation in the preparation of antisaccades and RT was found in both groups (Table 2).

We further asked whether changes in pupil size in the preparation of prosaccades would be associated with cognitive performance, symptom severity, and medication in individuals with schizophrenia. We did not observe any significant associations between pupil dilation in prosaccade preparation and cognitive performance, symptom severity, performance, and medication in individuals with schizophrenia. Furthermore, for both groups, we found no significant correlations between pupil dilation in prosaccade preparation and task performance measures (Supplementary Table S2).

## 4. Discussion

In this study, we investigated the relationship between changes in noradrenergic response, as measured by task-evoked pupil dilation, and cognition and symptomatology severity in individuals with schizophrenia. We found that preparatory pupil dilation in antisaccade trials was significantly reduced in schizophrenia outpatients as compared to healthy subjects, which was found to be associated with the severity of clinical symptoms (PANSS total score).

First, we Start date of the program for which the scholarship is requested (year/month)confirmed the hypothesis that individuals with schizophrenia have reduced task-evoked pupil dilation with increasing task demands compared to healthy subjects, which is consistent with the literature (32, 42–44, 48). In our study, individuals with schizophrenia were not able to increase their noradrenergic response to correctly perform a task that requires higher cognitive effort (i.e., antisaccade task), as demonstrated by reduced pupil dilation in task preparation. Surprisingly, the allocation of cognitive effort in antisaccade trials was not associated with better cognitive performance in any of the MATRICS domains, as opposed to the literature. For example, studies using a cognitive effort-based decision-making task observed that in individuals with schizophrenia, greater pupillary change on the hard versus easy conditions was correlated with better cognitive ability. However, these two studies did not include a healthy comparison group and the schizophrenia group was composed of participants with moderate to serious negative symptoms, reducing the generalizability of the findings (49, 57).

Additionally, reduced task-evoked pupil dilation in antisaccade preparations was significantly associated with higher PANSS total and, at a trend level, with higher PANSS negative scores. As cognitive deficits, negative symptoms correlate strongly with functional outcomes and respond insufficiently to conventional treatments (58). Previous studies reported that higher negative symptoms were associated with smaller pupil dilation (48, 49). Individuals with schizophrenia, particularly those with more severe negative symptoms, are less willing to exert appropriate effort, suggesting that they may overestimate the cost of difficult actions, which leads to avolition. Consequently, it may reflect reduced pupil dilation in tasks that require greater cognitive effort (e.g., antisaccade task).

The LC projects to various cortical regions, including the prefrontal cortex (PFC). Disruptions in LC-NA projections to the PFC and other brain regions have been linked to cognitive abnormalities (2, 59–61). In their study, Devilbiss et al. identified two distinct modes of LC activation: Phasic activation, driven by top-down attentional processing, and Tonic activation, associated with the maintenance of overall arousal and preparedness to respond to stimuli (62). Changes in pupil diameter serve as a reflection of LC activity and task-related cognitive demands, with increased pupil size indicating higher cognitive effort allocation and vice versa (26). Our study revealed that individuals with schizophrenia allocated significantly fewer cognitive resources during antisaccade trials, as evident from reduced pupil dilation, indicating insufficient effort allocation, which aligns with previous research (44, 49, 57). We propose that the observed reduction in pupil dilation among individuals with schizophrenia during the antisaccade task may be linked to dysregulated phasic LC activation.

What mechanisms might account for the relationship between dysregulated noradrenergic responses and schizophrenia symptom severity? Dysregulated noradrenergic pathways underlie schizophrenia symptoms, including both positive and negative manifestations (2). For instance, positive symptoms may result from hyper-vigilant states of consciousness, while negative symptoms could be linked to hypo-vigilant states (1). Positron emission tomography studies identified hyper-activation of the temporal cortex and limbic areas in positive symptomatology, along with hypo-activation of prefrontal areas in negative symptomatology (63, 64). Furthermore, chronic paranoid individuals exhibited elevated NA levels in the forebrain, particularly in the limbic region, as confirmed by postmortem investigations (21, 65, 66). These findings align with studies detecting increased NA and its metabolites in the CSF and plasma of individuals with schizophrenia, with a pronounced impact on paranoid subgroups (18, 41). Moreover, van Kammen et al. rigorously confirmed the link between noradrenergic activity and the exacerbation of both positive and negative schizophrenia symptoms through CSF measurements (67). In summary, these findings strongly support the close association between noradrenergic function and the manifestation of schizophrenia symptoms.

The present study includes some limitations. First, pupillary responses are an indirect measure of LC activity; and other neuromodulators (e.g., acetylcholine) along with NA may contribute to changes in pupil size (68). Second, all patients were using antipsychotic medications, which have anticholinergic properties that can influence pupil size (69, 70), although we did not observe any association between reduced pupil dilation and chlorpromazine and benztropine equivalents. Third, pupil dilation involves a circuitry of both top-down and bottom-up control, potentially implicating other brain regions associated with arousal and attention, such as the superior colliculus and frontal/prefrontal cortex (68, 71). For instance, microstimulation of the intermediate layer of the superior colliculus induces temporary pupil dilation in non-human primates (54). Nevertheless, the LC is considered pivotal for pupil dilation in response to cognitive effort, and dysregulation of the LC-NA system may contribute to schizophrenia symptom severity. Lastly, this study comprises a relatively small sample size. Future studies should investigate changes in pupillary responses in a population of unmedicated individuals and with a larger sample size. Moreover, studies focused on the role of each neuromodulatory circuit in the modulation of pupil size are still needed.

In conclusion, our findings suggest that the modulation of LC activity when cognitive demand increases is associated with overall symptom severity in individuals with schizophrenia. These findings shed further light on the mechanisms underlying schizophrenia pathophysiology. More importantly, the modulation of noradrenergic responses may provide a strategy to improve clinical symptoms in schizophrenia.

## Data availability statement

The original contributions presented in the study are included in the article/Supplementary material, further inquiries can be directed to the corresponding author.

## Ethics statement

The studies involving humans were approved by Brazilian National Committee of Ethics in Research (12990013.0.0000.5263) and pre-registered at ClinicalTrials.gov (1R03TW009002–01). The studies were conducted in accordance with the local legislation and institutional requirements. The participants provided their written informed consent to participate in this study.

## Author contributions

AP and RP conceptualized the study. AP was responsible for manuscript writing and conducted all the statistical analyses. AP and AG were responsible for subsequent revisions. RP oversaw project development and provided data interpretation. AP, AG, WS, NE, and LS collected data and provided data interpretation. All authors contributed to the article and approved the submitted version.
