# Supplementary material for: Dysregulated noradrenergic response is associated with symptom severity in individuals with schizophrenia
Source: Front Psychiatry. 2023 Nov 7;14:1190329. doi: 10.3389/fpsyt.2023.1190329 (PMC10661901; doi:10.3389/fpsyt.2023.1190329)
Supplement: Supplementary file 1 [file Data_Sheet_1.docx]

**SUPPLEMENTARY MATERIAL**

**Methods**

Converting arbitrary units to millimeters:

The EyeLink 1000 provides pupil area or diameter in arbitrary units (i.e. the units reflect the number of thresholded pixels). We simply followed the SR Research suggestion (details can be found in [Webinar - Recording and Analysing Pupil Data (Pupillometry)](https://www.youtube.com/watch?v=xaMc-8bU7Zc&list=PLOdF-B36TwsoXQh0UYuO4D__RzTLtPxX5&index=18) converting the arbitrary units (in that case arbitrary *area* units) into absolute units (i.e. millimeters).

Steps:

1. We printed out a black dot of a known size (we used 6 mm) on a piece of paper using a laser printer.
2. We attached the target to the chinrest equipment at the same distance as used for the subjects.
3. We recorded the black dot for a few seconds. Once we had recorded the file. we had the pupil size for the printed dot.
4. Finally. as we recorded the subject's pupil size data in AREA. we used a formula below to convert arbitrary units into mm.

For example:

The printed black dot had a area of 6 mm. If the eye tracker is reporting 7070 and if you put a real eye in the chinrest at the same distance as the black dot (i.e. 56cm) in your recording. and the eye tracker reports a diameter of 6000 then you can take the eye data to correspond to about 5.49mm. (because [6/sqrt(7070)] = [x/sqrt(6000)]).

**Results**

| **Supplementary table 1.** Results of the normality test. | |
| --- | --- |
| Shapiro-Wilk test (alpha=0.05) | |
| Speed of processing | W= 0.88  p= 0.01 |
| Visual learning | W= 0.87  p=0.009 |
| Attention | W= 0.96  p= 0.47 |
| Working memory | W= 0.95  p= 0.37 |
| Verbal learning | W= 0.96  p= 0.69 |
| Reasoning and problem solving | W= 0.88  p= 0.01 |
| Social cognition | W= 0.79  p= 0.0008 |
| Global cognition | W= 0.90  p= 0.03 |
| PANSS total | W= 0.96  p= 0.47 |
| PANSS positive | W= 0.74  p<0.0001 |
| PANSS negative | W= 0.88  p= 0.01 |
| PANSS general | W= 0.94  p= 0.26 |
| Antisaccade error rate | HC: W= 0.94, p= 0.13  SCZ: W= 0.90, p= 0.04 |
| Prosaccade RT (ms) | HC: W= 0.92, p= 0.06  SCZ: W= 0.91, p=0.05 |
| Antisaccade RT (ms) | HC: W= 0.90, p= 0.07  SCZ: W= 0.95, p=0.31 |
| Baseline pupil size (mm) | Prosaccade: HC: W= 0.94, p= 0.18; SCZ: W= 0.95, p= 0.31  Antisaccade: HC: W= 0.95, p= 0.19; SCZ: W= 0.93, p= 0.15 |
| Pupil dilation in antisaccade preparations (mm) | HC: W= 0.97, p= 0.67;  SCZ: W=0.93, p= 0.12 |
| Pupil dilation in prosaccade preparations (mm) | HC: W=0.96 , p=0.48;  SCZ: W= 0.92 , p= 0.16 |
| Age in years | HC: W= 0.95, p= 0.29;  SCZ: W= 0.95, p=0.38 |
| CPZ equivalent | W= 0.84, p= 0.001 |
| Benztropine equivalent | W= 0.80, p= 0.007 |

HC, healthy controls; SCZ, individuals with schizophrenia; CPZ, chlorpromazine

| **Supplementary table 2**. Associations between change in pupil size in prosaccade preparation. cognition. clinical symptoms and medication in individuals with schizophrenia. | | | |
| --- | --- | --- | --- |
|  | Change in pupil size (mm) | | |
|  | r | p | N |
| Cognition (z-score) |  |  |  |
| Global cognition | 0.20 | 0.36 | 23 |
| Attention | -0.11 | 0.58 | 23 |
| Speed of processing | 0.15 | 0.47 | 23 |
| Working memory | 0.12 | 0.57 | 23 |
| Visual memory and learning | 0.27 | 0.90 | 23 |
| Verbal memory and learning | 0.24 | 0.27 | 23 |
| Reasoning and problem solving | -0.13 | 0.53 | 23 |
| Social cognition | 0.18 | 0.43 | 20 |
| Clinical symptoms |  |  |  |
| PANSS total score | -0.16 | 0.46 | 23 |
| PANSS positive score | 0.05 | 0.81 | 23 |
| PANSS negative score | -0.09 | 0.65 | 23 |
| PANSS general score | -0.01 | 0.93 | 23 |
| Performance |  |  |  |
| Prosaccade error rate | -0.29 | 0.17 | 23 |
| Prosaccade RT (ms) | -0.13 | 0.53 | 23 |
| Medication (mg) |  |  |  |
| CPZ equivalent | 0.15 | 0.47 | 23 |
| Benztropine equivalent | 0.28 | 0.21 | 21 |

PANSS. Positive and Negative Syndrome Scale. CPZ. chlorpromazine. RT. reaction time. r and p values of the Pearson or Spearman correlations (2-tailed).

*Pupil dilation in task preparation after exclusion of blinks.*

The effect of trial (prosaccade/antisaccade) and group on pupil dilation was assessed using a mixed model ANOVA. There was a significant main effect of trial condition (F[1, 49] = 11.00 p = 0.002, η2= .18), trial-by-group interaction (F[1, 49] = 11.49, p = 0.001, η2= .19) and a trend towards significance for group (F[1, 49] = 4.65, p = 0.06, η2= .09).
